# A Systematic Review of Interventions Related to Body Awareness in Childhood

**DOI:** 10.3390/ijerph19158900

**Published:** 2022-07-22

**Authors:** Si Nae Ahn

**Affiliations:** Department of Occupational Therapy, Cheongju University, Cheongju 28503, Korea; otlovesn@cju.ac.kr

**Keywords:** body perception, body schema, child, motor skills, sensorimotor defects

## Abstract

Body image involves perceptions, attitudes, and beliefs concerning one’s body, while body schema involves the sensorimotor capacities to control movement and posture. A review study is needed to obtain sufficient data to determine the effect of body awareness on the normal development of children. This study is a systematic review of interventions related to body awareness among typically developing children. Studies focusing on interventions related to body awareness from 2010 to 2021 were analyzed and extracted from four major scientific databases, and the three that met the inclusion criteria were analyzed. Methods were analyzed by frequency, and the results were analyzed by calculating effect sizes. Two studies involved a nonrandomized two-group design and a single-case experimental study. Two studies targeted early childhood, and one study focused on middle childhood. This review indicates that determining the effectiveness of interventions related to body awareness is necessary for typically developing children because body awareness is a potential influencing factor in their development. Additional research is needed to determine the effectiveness of body awareness assessment tools and interventions.

## 1. Introduction

The American Psychological Association Dictionary of Psychology defines body schema as the cognitive organization of one’s appearance, including internal image, thoughts, and feelings [1]. The Merriam-Webster Dictionary describes schema as a diagrammatic presentation or a structured framework or plan (e.g., an outline) and a mental codification of experience that includes a particular organized way of cognitively perceiving and responding to a complex situation or set of stimuli [2]. The body schema is regarded as individuals’ cognitive perceptions and representations of their whole human body [3], whereas Dumont [4] defined body schema as the motor–tactile–kinesthetic awareness of one’s own body. Slaughter and Heron [3] hypothesized that there are three levels of the early development of human body knowledge—sensorimotor, visuospatial, and lexical semantic. Sensorimotor body knowledge governs conscious knowledge of children’s own bodies; visuospatial body knowledge consists of their ability to point to and name their specific body parts; and lexical semantic body knowledge refers to their language-based knowledge of the functions and locations of their body parts. The concept of body knowledge is also known as body perception or body awareness [5]. Physical self-concept, a concept related to body schema, is seen as a multidimensional subdomain of the overall self-concept that incorporates various traits, such as health, appearance, and physical activity [6]. Consequently, physical self-concept has been considered to play an important role in children’s health. Physical self-concept is of great relevance owing to its effect on children’s level of physical activity [7,8,9] and health, leisure, and social relationships [10,11].

This structured framework of the human body facilitates the development of children’s body awareness—that is, their conscious knowledge of their own bodies in the external world—and refers to the information they first obtain through sensory impressions, such as the sensory modality of vision. These multisensory processes are key to the development of children’s body perceptions and representations and the formation of links between the layout of their limbs, their body positioning, body movements, and visual experiences. Simons et al. [5] suggested that this relationship involves children’s perceptions and representations of their (a) physical appearance; (b) purposeful bodily movements (e.g., direction, performance, and intensity); (c) positioning of bodies and body parts, both in space and toward each other; and (d) perception of their bodies in relation to the external environment (e.g., through somatosensory, auditory, and visual information). Njiokiktjien et al. [12] posited that children develop body awareness through interactive psychomotor expression (i.e., body language)—that is, by watching others and forming a perspective of themselves in relation to others.

Woertman [13] linked the development of individuals’ body consciousness to the emergence of their consciousness of the external world. Simons et al. [5] postulated that the consciousness of individuals’ own body and body parts includes their body postures, positions, and movements and is accompanied by their awareness of their bodies’ laterality and directionality and the tension and relaxation of their breath. Scholars have defined awareness as individuals’ ability to organize new stimuli and incorporate them into previously stored information and, in the motor domain, have associated this awareness with individuals’ sensorial integration, interpretation, activation, and reinforcing capabilities. Hence, scholars consider body awareness the product of the interrelationship among individuals’ neurologic and behavioral aspects that integrate their bodies and the external environment, as well as the basis of individuals’ psychomotor structure [14].

Body awareness develops primarily through children’s motor experiences from infancy [15,16]. Developmentally, from the age of 24 months onward, children become more aware of both their body and its capabilities, and this translates into more purposeful movements. As children mature, their body awareness progresses at a more rapid and intense pace and plays a key role in their behavior as they learn new movements and acquire new knowledge [5].

Bertoldi et al. [14] found that children with chronic movement problems lack autonomy in problem solving involving movement restrictions and suggested that one of the factors contributing to these problems is their inadequate development of body awareness. Consequently, they proposed the creation of activities that are playful, challenging, and rich in a variety of physical behaviors in the early stages of children’s development. They also suggested the integration of different disciplines to promote the development of body cognition in children with motor deficits. Vallaey and Vandroemme [17] noted that delays in movement skill development and body consciousness can carry significant negative consequences for individuals’ overall development.

Based on these prior findings, body awareness is highly related to children’s development. However, although scholars have studied the effectiveness of treatments that include the element of body awareness in various diagnoses, there is scant research related to body recognition among children. In particular, to obtain sufficient data to determine the appropriate treatment in the rehabilitation process of children with developmental delays, scholars should evaluate the level of children’s body recognition ability in relation to their developmental stage. As few studies provide evidence through an in-depth examination of children’s body perceptions, further research is needed.

This study provides comprehensive evidence by analyzing the effectiveness of interventions related to body awareness among typically developing children through a systematic review of the literature.

## 2. Materials and Methods

### 2.1. Review Design

**Figure 1:**
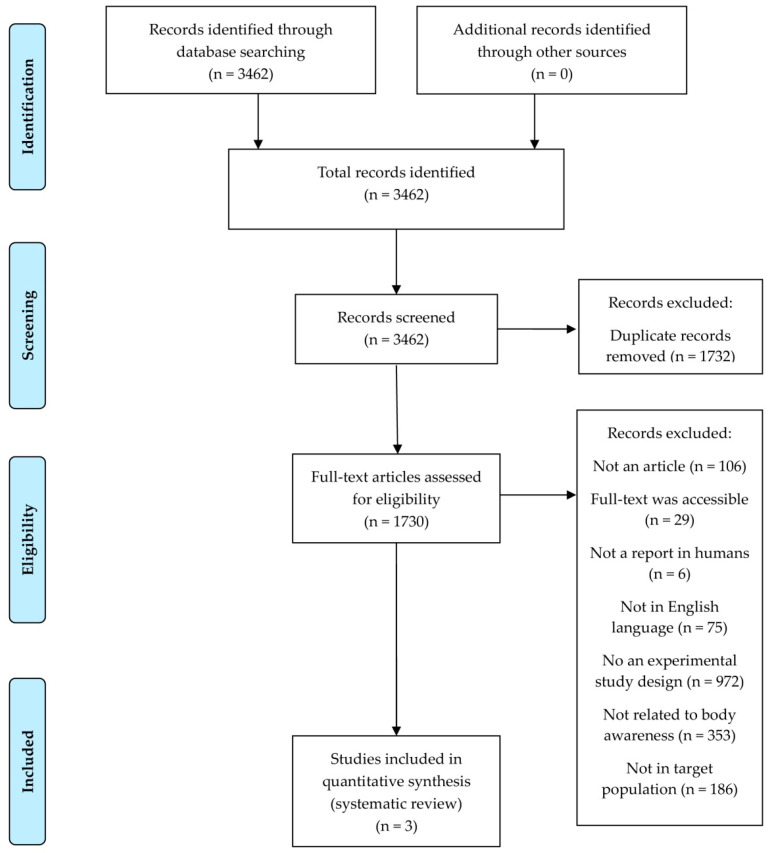
PRISMA flow diagram.

This study systematically reviewed the assessment tools and interventions related to body awareness in preschool- and school-aged children following the guidelines of the 2020 Preferred Reporting Items for Systematic Reviews and Meta-Analyses (PRISMA) Statement [18]. The PRISMA flow diagram is presented in Figure 1. This review was conducted with reference to the criteria developed for systematic reviews outlined in the A MeaSurement Tool to Assess systematic Reviews (AMSTAR) checklist [19].

### 2.2. Search Strategy

Articles published between January 2010 and November 2021 were reviewed. The search strategy retrieved items from four scientific databases: Academic Search Ultimate, MEDLINE, PubMed, and Web of Science. The search string used in all databases was exported as “body awareness” or “physical awareness” or “interceptive awareness” based on inclusion and exclusion criteria.

### 2.3. Study Selection Process

The inclusion criteria were as follows: studies written in English retrieved in full text, studies aimed at childhood, studies including experimental study designs, and studies related to the interventions of body awareness. The exclusion criteria were research on animals, studies that did not target typically developing children according to ICD-11 disease classification criteria, study designs that did not include interventions, and studies that did not include interventions related to body awareness. Only original studies published in peer-reviewed journals were considered.

Original articles that matched the inclusion and exclusion criteria for this study were extracted from the four databases. A total of 3462 records were identified by inserting keywords into the database. After retrieving the studies, duplicates were removed after examination of the studies’ titles. Studies’ abstracts were reviewed using the inclusion and exclusion criteria. For studies that could not be categorized according to the inclusion and exclusion criteria based on their abstracts, a full-text review was conducted. The final systematic review included three studies that met the eligibility criteria.

### 2.4. Risk of Bias

The Downs and Black scale was used to assess the risk of bias. The Downs and Black checklist consists of 27 questions related to quality of reporting, external validity, internal validity (bias and confounding), and statistical power [20]. In the reporting part of this scale, the maximum score for the items investigating the distributions of principal confounders is 2, and all other items have a score of 1 or 0. This scale contains a maximum total score of 28 points. The Downs and Black scores were given corresponding quality levels of 26–28 (excellent), 20–25 (good), 15–19 (fair), and ≤14 (poor) [21].

### 2.5. Data Extraction and Analysis

The collected data were extracted from the final three studies that met the eligibility criteria. The variables extracted were as follows: author, year of publication, size of study population, assessment tools and interventions related to body awareness, and intervention periods.

Inclusion criteria included experimental study designs; studies were classified using the five stages of evidence-based classification presented by Arbesman and Lieberman [22]: Level I study: a randomized controlled trial; Level II study: nonrandomized assignments to treatment or control; Level III study: without controls; Level IV study: single-case experimental study design; and Level V study: case report. In addition, the evidence in all studies is presented according to the patient/population problem, intervention, comparison, and outcome (PICO) process of evidence organization.

The results of interventions related to body awareness were examined and used to calculate effect size (biserial d). The results are presented as frequencies according to children’s ages [23].

## 3. Results

### 3.1. Study Selection

A database search identified 3462 studies, but after review, 1732 duplicate studies were excluded. Among these, 106 non-original studies were eliminated, and the remaining 1701 studies were assessed for eligibility upon review of the abstract and full text. Per the inclusion and exclusion criteria, 1624 studies were dismissed for the following reasons: studies in which the full text was not provided (n = 29), studies that did not contain reports on humans (n = 6), studies that were not in the English language (n = 75), studies that were not experimental study designs (n = 972), studies that were not related to body awareness (n = 353), and studies whose study participants were not the target population (n = 186). Three studies met the eligibility criteria and were consequently included in the data extraction. The study selection process is summarized in the flowchart in Figure 1.

### 3.2. Study Design

A variety of study designs were used in this systematic review. The quality of evidence of the selected studies was evaluated using evidence-based levels of classification. The methodological quality was analyzed by applying the evidence-based, five-stage classification suggested by Arbesman and Lieberman [22]. Each study included a nonrandomized two-group design, a nonrandomized one-group design, or a single-case experimental study (Table 1).

### 3.3. Risk of Bias of Included Studies

The risk of bias in the studies that were included was assessed using the Downs and Black scale. Quality scores included 26–28 (excellent), 20–25 (good), 15–19 (fair), and ≤14 (poor). In this review, the overall risk of bias in the included studies was assessed as fair to poor. One study was rated as being of poor quality, while two studies were rated as fair (Table 2).

### 3.4. General Characteristics of Studies

The evidence of all the studies is presented according to the PICO process of evidence organization. The sample of the three studies comprised 1406 participants; the average number of participants was 468.7 ± 509.6 (range = 33–1029) per study; and the participants’ age range was 3–11 years. The mean treatment duration was 53.3 ± 11.5 (range = 40–60) minutes, and the mean number of treatment sessions was 21.3 ± 15.1 (range 4–32). Two studies targeted early childhood [24,25], and one study targeted middle childhood [26] (Table 3).

### 3.5. Results of Effectiveness of Interventions Related to Body Awareness

Analyses were performed on the three studies included in this review to present the required statistical data. All three studies reported an increase in the primary outcome measurement post-intervention (Table 4). Halliwell et al. [26] reported an increase in utilizing the Body Appreciation Scale-2 for Children after a yoga-based body awareness intervention. Pienaar et al. [25] reported an increase in the perceptual–motor skills of these preschoolers that contributed to school readiness at the attention and cognitive levels after a body awareness intervention. That study examined the effect of a Kinderkinetics program, which was provided as an intervention method related to body awareness, on components of children’s perceptual–motor and cognitive development. The program interventions included fundamental locomotor skills, stability, manipulation, and bilateral integration activities. Body awareness; spatial orientation; balance; general, hand–eye, and foot–eye coordination; fundamental skills; and activities for vestibular stimulation were then addressed, after which the lesson ended with an enjoyable activity. All of these skills and abilities are considered important perceptual and motor requirements for the development of basic learning skills in children. The Peabody Developmental Motor Scales-2 was used as an evaluation tool to measure the effectiveness of intervention-related body awareness [25]. Battaglia et al. [24] also reported an increase. In that study, a physical education program was provided as an intervention method related to body awareness. The interventions included in the program were lucid motor activities aimed at developing body awareness and fundamental motor and perceptual–sensory skills. The quotient of gross motor development was used as an evaluation tool to measure the effectiveness of intervention-related body awareness [24].

## 4. Discussion

The aim of this systematic review was to comprehensively examine evidence on the effectiveness of interventions related to body awareness among typically developing children. Three studies comprising more than 1400 participants were incorporated in this review. Two studies involved a nonrandomized two-group design and a single-case experimental study. All of the selected studies reported increased body awareness post-intervention [24,25,26]. However, an analysis of the level of evidence in the selected studies showed that one study was rated as poor quality, and two studies were rated as fair. It may be difficult to clearly interpret the effectiveness of interventions related to body awareness because the selected studies had low-quality evidence.

Body awareness begins early in a child’s life and is unconscious and considered involuntary. As development continues, the child can point to body parts and then later use vocabulary for those body parts and their function. Children’s further growth and positive development are aided by an understanding of their body, allowing them to use it to develop more complex movement activities [27]. First, previous research indicates that early childhood is characterized by reasoning about one’s own body as an object. Early childhood showed better-than-chance performance when deciding which of two apertures was the appropriate size for their body. The reported interpretive relation between this performance and body awareness supports the interpretation that these are components of a larger suite of self-related competencies contributing to a coherent developmental trajectory. As children move more independently through their everyday physical and social world, their developing body awareness is reflected in their movements [28]. Next, middle childhood is understood as the continuity of self over time. Previous research has shown that as children develop, there is a link between children’s grasp of their body as a unique object in the environment and their grasp of their mind as a unique manipulator. Converging evidence for such a link indicates difficulties with body recognition in children with neurodevelopmental disorders, such as awareness of their body in space, irregularities in mirror self-recognition, and self–other source memory [29,30,31].

This review identified an increase in body awareness of certain attributes in typically developing children post-intervention [24,25,26]; however, scholars have conducted few studies of children with various neurodevelopmental disorders. Additionally, different objective measurements of body awareness were used across the three studies. This suggests that there remains a lack of common assessment instruments ensuring the transferability of findings between studies to represent body awareness in children. More evidence is needed from studies of children with disabilities.

In this review, several studies included a small number of participants, limited convenience samples, and decreased evidence levels of experimental study designs; one study was rated as poor quality, and two studies were rated as fair, which may have reduced the generalizability of the results. Therefore, these studies require further development—such as the inclusion of a comparison against a control group and the implementation of the intervention in a larger sample size—before their effectiveness can be fully evaluated.

Previous studies’ findings have suggested that body awareness may be useful in the treatment of individuals with chronic diseases, such as chronic low back pain and chronic renal failure. The findings of this systematic review suggest that body awareness treatment should include desirable features for rehabilitation, meaningful and motivational elements, and adaptive feedback to increase body recognition capabilities among typically developing children. This systematic review demonstrated that body awareness therapy is feasible and effective. Furthermore, the recent increase in the number of studies related to body awareness in childhood reflects the trend of increasing interest in children’s body awareness. Although this study confirmed both the necessity and the practical possibility of teaching children to develop body awareness, more evidence is needed to reveal the positive effects of childhood body awareness intervention methods and confirm the validity of the appropriate assessment tools.

### Limitations

This systematic review had several limitations. The available literature included only a small number of published studies, and those studies exhibit heterogeneity in study methodology design and quality. As detailed descriptions were not provided for direct comparison of each study, the general characteristics of the selected articles are provided in a table for comparison. In addition, the generalizability of the results is limited owing to the small sample sizes and failure to use probabilistic sampling methods. To compensate for these limitations, further research should be conducted to yield more extensive studies related to body awareness using various keywords and diverse databases.

## 5. Conclusions

This systematic review comprehensively examined evidence on the effect of interventions related to body awareness among typically developing children. These findings summarize the general characteristics of the overall intervention types and provide a source of evidence. This systematic review confirms that children’s body awareness is important because it can positively influence development among typically developing children. The objective effectiveness should be verified in a meta-analysis.

## Figures and Tables

**Table 1:** Number of Selected Articles by Level of Evidence.

Levels of Quality	Definition	Frequency (%)
I	Randomized, controlled trials	0 (0.0)
II	Nonrandomized two-group studies	2 (66.6)
III	Nonrandomized one-group studies	0 (0.0)
IV	Single-case experimental studies	1 (33.3)
V	Case reports	0 (0.0)
Total		3 (100.0)

**Table 2:** Risk of Bias Assessment.

Author (Year)	Downs and Black Scale (Total Score)					
	Reporting	External Validity	Internal Validity–Bias	Internal Validity–Confounding (Selection Bias)	Power	Sum of Score
Battaglia et al. [24]	7 (11)	3 (3)	4 (7)	1 (6)	1 (1)	15 (28)
Pienaar et al. [25]	7 (11)	0 (3)	5 (7)	2 (6)	0 (1)	14 (28)
Halliwell et al. [26]	6 (11)	3 (3)	5 (7)	4 (6)	1 (1)	19 (28)

**Table 3:** Evidence Related to Body Awareness for Children (n = 3).

Author	Level of Evidence/ Participants/ Age Classification	Intervention		Outcome Measurements	Outcome
		Group	Session/Time		
Battaglia et al. [24]	Level IV n = 1029 Early childhood (3–5 years)	Experimental group: Physical education program	60 min, twice per week, 16 weeks	Body mass index; quotient of gross motor development; preliteracy skills	The physical education program improved motor and cognitive learning in preschoolers and can be a definitive educational strategy for achieving successful academic outcomes.
Pienaar et al. [25]	Level II n = 33 Early childhood (4–6 years) Experimental group: n = 13 Control group: n = 20	Experimental group: Kinderkinetics program Control group: No intervention	1 h, once per week, 7 months	Peabody Developmental Motor Scales-2; Junior South African Individual Scale	The Kinderkinetics program was effective in improving the perceptual–motor skills of these preschoolers and contributed to school readiness at the attention and cognitive levels.
Halliwell et al. [26]	Level II n = 344 Middle childhood (9–11 years) Experimental group: n = 190 Control group: n = 154	Experimental group: Yoga Control group: Physical education	40 min, once per week, 4 weeks	Body Esteem Scale for Children; Objectified Body Consciousness Scale–Youth; Body Appreciation Scale-2; Positive and Negative Affect Scale for Children	The yoga intervention group evaluated the sessions very favorably.

**Table 4:** Effectiveness of interventions related to body awareness.

Author	Assessment Tools	Biserial d	95% Confidence Interval	Variable	Significant
Battaglia et al. [24]	Quotient of gross motor development	0.2328	0.0942 to 0.3714	0.005003	Significant
Pienaar et al. [25]	Peabody Developmental Motor Scales-2	3.2845	1.16184 to 4.9506	0.72261	Significant
Halliwell et al. [26]	Body Appreciation Scale-2 for Children	0.4228	0.1353 to 0.7104	0.021523	Significant

## Data Availability

The systematic review data used to support the findings of this study are included within the article.
